# Some Trade Unionists on Doctors

**Published:** 1911-09-23

**Authors:** 


### The Hospital

A Journal of

The Medical Sciences and Hospital Administration.

New Series. No. 243, Vol. IX. [No. 1315, Vol. L.] SATURDAY, SEPTEMBER 23, 1911.

### Some Trade Unionists on Doctors.

It is one of the weaknesses—or, to preserve the philosophic attitude, let us say characteristics—of human nature that it cannot stomach very well submitting to authority. Although to inculcate the social necessity of such submission is one great object of all education, nobody of any independence, high or low, likes it; for the aristocratic hatred of experts is just as common as the democratic. And of those who profess to like it, who profess cheerfulness in deferring their own judgment, a good number are humbugs. But a small minority gets round the difficulty by a shorter and less irksome way. These self-confident folk take their own prejudice as the voice of reason, and proceed to vilify the authority which commits the crime of seeming to put them in the wrong. The fault is, almost unavoidably, part and parcel of the make-up of the revolutionist temperament. It accounts, for instance, for Tolstoi's hatred of doctors. In " Anna Karenina " he expatiates on the brutality of one of them who, having to examine a young lady's chest, insisted upon the removal of the garments covering that part of her person. Tolstoi simply could not forgive medical men for the right special knowledge gives them to override certain susceptibilities. Really, lay critics are hard to please, for recently some of them have been saying club doctors are negligent enough to apply the stethoscope to their patients' clothes! This, however, by the way. The point is that if a person has passed tests placing him in a position to judge, the unqualified have no right to deny the privilege, and, if they contend for the right, simply show themselves possessed by a childish objection to reasonable direction.

Tolstoi was a man of genius and may be forgiven much. Speakers at trade union conferences are scarcely that, but as recent events, upon which no opinion is expressed, have placed them in a position of considerable prominence, it will be well, they having just made a virulent attack upon the medical profession, to have at the outset defined before us, from the determinist standpoint, probably the chief motive in leading them to spout mischevious nonsense. The charge made at the recent Trades Union Congress is of venality of the most serious and criminal kind. It is asserted that medical men, in the supposed interest of employers of labour, commonly return as fit for work those they know to be seriously ill or even dying. The violence of the charge, it will be seen at once, quite rebuts it. If anything of this kind had been going on, a good many people would have got hold of it very quickly. Take as an actual instance the last annual report of one of the large medical protection societies which undertake the defence of medical men in legal and other affairs. Most of the cases reported are those where persons who had made charges against doctors showed themselves anxious to drop them upon further consideration of the matter. Of justification for this libel there is simply no vestige to be found. It would be absurd, indeed, to notice such a thing, were it not for the large numbers who now read popular newspapers and believe all they see, and want nothing more. If the accusers—we assume their personal good faith in advancing this gross accusation—had, besides Tolstoi's revolutionary spirit, the smallest fraction of Tolstoi's knowledge of the human heart, they would know that villainy such as this is rare anywhere, and that although mercenary snobs are known, it would be hard to find a body of men who, from the scientific, non-political nature of their calling, and its neutral benevolence, are freer from them than the medical profession.

One does not expect the *suaviter in modo* from those whose hard lot has left them little leisure for the graces of life, but nothing could soften the meaning here expressed. In all probability this giving the rein and spur to prejudice may be correctly diagnosed as a piece of bullying, and as such is greatly to be regretted. One had thought that in the great modern democratic movement medical men could play in their own sphere a useful part. If, however, charges of this kind, minus any detail of proof, receive cheers and only a single protest, strong doubts begin to suggest themselves. It must be remembered, however, that the atmosphere of the meeting was at the time rather electrical, largely by reason of preliminary fisticuffs. In less emotionally charged surroundings, common-sense would surely have scouted the bogey. None the less, there is a useful lesson as to the need for alert combination, and existing medical organisations will take due note. There is no doubt high-handed usage by extremists can be checked, if one may judge from the history of past medical protests. But we wish to speak in no provocative spirit. If Bismarck had at last to go to Canossa and retract his defiance of a learned profession, let us hope the "strike leaders" will take the next black eye, with fair words instead of foul, to the surgery door—where they will receive every possible attention.

